# Enhancing the Morpho-Structural Stability of FAPbBr_3_ Solar Cells via 2D Nanoscale Layer Passivation of the Perovskite Interface: An In-Situ XRD Study

**DOI:** 10.3390/nano15050327

**Published:** 2025-02-20

**Authors:** Barbara Paci, Flavia Righi Riva, Amanda Generosi, Marco Guaragno, Jessica Barichello, Fabio Matteocci, Aldo Di Carlo

**Affiliations:** 1 Spec-X Lab, Istituto di Struttura della Materia Consiglio Nazionale delle Ricerche, Via del Fosso del Cavaliere 100, 00133 Roma, Italy; flavia.righiriva@artov.ism.cnr.it (F.R.R.); marco.guaragno@artov.ism.cnr.it (M.G.); aldo.dicarlo@artov.ism.cnr.it (A.D.C.); 2 CHOSE—Centre for Hybrid and Organic Solar Energy, Department of Electronic Engineering, University of Rome “Tor Vergata”, Via del Politecnico 1, 00133 Roma, Italy; jessica.barichello@artov.ism.cnr.it (J.B.); fabio.matteocci@uniroma2.it (F.M.)

**Keywords:** formamidinium lead bromide perovskite solar cells, 2D passivation, morpho-structural stability, in situ XRD

## Abstract

Despite the huge progress achieved in the optimization of perovskite solar cell (PSC) performance, stability remains a limiting factor for technological commercialization. Here, a study on the photovoltaic, structural and morphological stability of semi-transparent formamidinium lead bromide-based PSCs is presented. This work focuses on the positive role of 2D nanoscale layer passivation, induced by perovskite surface treatment with a mixture of iso-Pentylammonium chloride (ISO) and neo-Pentylammonium chloride (NEO). In situ X-ray diffraction (XRD) is applied in combination with atomic force microscopy (AFM), and the results are correlated to the devices’ photovoltaic performances. The superior power conversion efficiency and overall stability of the ISO-NEO system is evidenced, as compared to the un-passivated device, under illumination in air. Furthermore, the role of the ISO-NEO treatments in increasing the morpho-structural stability is clarified as follows: a bulk effect resulting in a protective role against the loss in crystallinity of the perovskite 3D phase (observed only for the un-passivated device) and an interface effect, being the observed 2D phase crystallinity loss spatially localized at the interface with the 3D phase where a higher concentration of defects is expected. Importantly, the complete stability of the device is achieved with the passivated ISO-NEO-encapsulated device, allowing us to exclude the intrinsic degradation effects.

## 1. Introduction

PSCs are currently gaining increased interest for next generation photovoltaic applications as promising alternatives to silicon-based technology [1,2,3,4]. The optimization of ultra-low-cost solution-based methods for the fabrication of perovskite thin films, has indeed boosted the performances of PSCs, leading to great improvements, over the past decade, in device efficiency and stability [5,6,7,8,9,10,11,12,13]. Among the advantages that PSCs exhibit over the traditional photovoltaic technology, adjustable transparency and color tunability are two main characteristics making them suitable candidates, especially for applications requiring a high visible transmittance, such as building integrated photovoltaics (BIPV) [14,15,16,17,18]. The perovskites mostly employed for the realization of highly efficient devices include the hybrid organic–inorganic iodide-based compounds MAPbI_3_ and FAPbI_3_ (where MA = methylammonium, CH_3_NH_3_^+^ and FA = formamidinium, HC(NH_2_)_2_^+^). Despite the high performances achieved, especially for FAPbI_3_-based PSCs, a main limiting factor of iodide-based perovskite derives from its intrinsic instability due to its phase transition (from α to δ phase) tendency [19,20]. For this reason, several strategies have been developed for the optimization of stability and power conversion efficiencies (PCE) in FAPbI_3_-based PSCs, including the effective coating of the active material and compositional engineering. This latter approach has recently driven interest in the investigation of bromide-based perovskites; it was indeed found that FAPbBr_3_ exhibits a superior stability against degradation with respect to its iodide-based counterpart, FAPbI_3_ [21]. Thanks to its wide gap (2.3–2.4 eV), FAPbBr_3_ has gained particular interest as potential active material in multi-junction tandem solar cells [22,23] as well as semitransparent and flexible planar devices [24,25,26,27,28,29]. At present, the PCE of FAPbBr_3_-based solar cells has increased up to ~11% [30,31,32,33,34]. Strategies commonly employed to limit the energy loss in FAPbBr_3_ PSCs exploit the regulation of the perovskite/charge transport layer interface in order to decrease charge recombination and increase charge transport efficiency. Following this approach, Qian et al. demonstrated that the use of a buffer layer at the FAPbBr_3_/electron transporting layer (ETL) interface can effectively facilitate the charge transport, leading to a PCE of 8.02% and a Voc of 1.43 V [35]. An additional common strategy for increasing the PCE includes the optimization of the design of high-quality cubic crystal perovskite films by additive engineering, interface engineering and crystallization regulation. Zhang et al., for example, found that the use of urea added into the perovskite precursor solvent might help in tuning the intermolecular ion exchange, resulting in FAPbBr_3_ devices with a PCE of 10.61% and Voc = 1.56 V [34]. More recently, Xu et al. proposed the use of guanidinium bromide to modulate the crystallization process and heal charge defects in FAPbBr_3_ perovskite solar cells, with a PCE of 8.92% and Voc = 1.639 V [36]. Zhu and coworkers demonstrated that a PCE of 9.12% can be obtained in inverted PSCs by the effective management of the δ-FAPbBr_3_, achieved by phosphonate/phosphine oxide additives [33], and that an efficiency improvement up to 11.14% can be achieved thanks to the use of self-assembled monolayers of (2-(3,6-diiodo-9H-carbazol-9-yl)ethyl)phosphonic acid as hole selective contact [31]. The optimization of FAPbBr_3_ crystal growth by intermediate complexes can be obtained through intermediate-phase-transition-assisted processes resulting in highly efficient devices with PCE values up to 10.86% and V_OC_ = 1.57 V [17]. The passivation of interfacial defects by proper additives was also found to strongly enhance PSC performance [37,38,39]. Recently, evidence of an improvement in the performance and stability of FAPbBr_3_ devices was also observed, following surface treatments that led to the formation of a 2D–3D perovskite interface [40]. Liu et al. demonstrated that the treatment of the FAPbBr_3_ surface with phenetylammonium bromide (PEABr) lead to the formation of an ultra-thin layer of 2D PEA_2_PbBr_4_ perovskite on the surface of 3D FAPbBr_3_, with a consequent enhancement of the PCE from 7.7% to 9.4% [41]. This follows that the recent progresses in terms of efficiency are further boosting the interest on novel approaches for prolonging PSC stability.

In this context, here, a study on the morpho-structural stability of FAPbBr_3_-based PSCs under light stress is presented. The present work specially focuses on the role of surface passivation via the use of a mixture of the following two bulky cations from chloride salts: ISO and NEO. The devices investigated exploit a thin film of FAPbBr_3,_ sandwiched between a layer of poly[bis(4-phenyl)(2,4,6-trimethylphenyl)amine] (PTAA) and TiO_2_/SnO_2,_ as charge transporting layers. Fluorine-doped tin oxide glass (Glass/FTO) and Indium tin oxide (ITO) are used as the bottom and top electrodes, respectively. In situ XRD [42] and ex situ AFM [43] are used to investigate the structural and morphological changes in the material under prolonged light stress [43]. In order to evaluate the role of the 2D passivation induced by the ISO-NEO treatments on the morpho-structural stability of the active material, a parallel study was performed on two systems, correlating the morpho-structural properties to the photovoltaic performances as follows: the Glass/FTO/c-TiO_2_/SnO_2_/FAPbBr_3_/PTAA/ITO cell (considered as a reference) was compared to the Glass/FTO/c-TiO_2_/SnO_2_/FAPbBr_3_/ISO-NEO/PTAA/ITO ISO-NEO-passivated device. In addition, intermediate systems terminated with the FAPbBr_3_ film were studied for a more detailed inspection of the perovskite layer.

## 2. Materials and Methods

Device fabrication: Transparent conductive FTO substrates (2.2 mm-thick TEC15 Pilkington, 15 Ω/square), with a square shape of 25 × 25 mm^2^, were patterned to electrically isolate the substrate by using a nanosecond pulsed laser (Nd:YVO4, λ = 355 nm, 15 ns, pulsed at 80 kHz with a fluence per pulse of 715 mJ cm^−2^). The patterned substrates underwent a two-step cleaning process. First, they were mechanically washed with de-ionized water and soap (a 1:5 mixture of Hellmanex and water) to remove dust and organic contaminants. Subsequently, they were further cleaned using ultrasonic baths with de-ionized water and 2-propanol, each for 10 min at 40 °C. The 40 nm-thick TiO_2_ compact layer (c-TiO_2_) was deposited by spray pyrolysis at 460 °C, starting from a precursor solution made up of titanium diisopropoxide bis(acetylacetonate), acetylacetone and ethanol in a 3:2:45 volume ratio. Then, 2% Nb doping was made by adding niobium ethoxide (Sigma Aldrich, Saint Louis, MI, USA) to the precursor solution. SnO_2_ nanoparticle-based ink at 1:20 *v*/*v* in deionized water was deposited on the Nb: TiO_2_-coated substrate using spin coating at 4000 rpm for 20 s. Then, the substrates were annealed at 120 °C for 20 min. Prior to perovskite deposition, the annealed substrates were subjected to a UV–ozone treatment for 30 min. This treatment improved the wettability of the surface, leading to better perovskite film distribution. Substrates were then transferred to a nitrogen-filled glove box for the perovskite deposition. A stoichiometric perovskite solution (FAPbBr_3_) was prepared by mixing 1 M PbBr_2_ (TCI Chemical, Tokyo, Japan) and 1 M FaBr (Great Cell Materials, Queanbeyan, Australia) in the DMSO solvent. To enhance the film formation process, 40 µL/mL of BMIMBF4 (1-Butyl-3-methylimidazolium tetrafluoroborate, Sigma Aldrich) ionic liquid (IL) precursor was added to the FAPbBr_3_ solution from a stock solution of 50 µL/mL in DMSO. Then, the FAPbBr_3_ perovskite films were deposited by spin coating at 4000 rpm for 20 s on warm substrates (60 °C), using the solvent quenching method (200 µL of ethylacetate dropped after 10 s). Finally, the samples were annealed at 80 °C for 10 min. Solutions of NEO and ISO (GreatCell Materials) were prepared at a concentration of 1 mg/mL in 2-propanol. An ISO-NEO solution was then created by mixing equal volumes (1:1 *v*/*v*) of the prepared ISO and NEO stock solutions. All these solutions were spin-coated onto the substrates at 4000 rpm for 20 s. The PTAA solution was prepared by dissolving 10 mg of 10 kDa PTAA powder (Solaris Chem, Shinjuku City, Tokyo, Japan) in 1 mL of toluene. The solution was then doped with 10 µL/mL of 4-tert-butyl pyridine (TBP, 96%, Sigma Aldrich) and 5 µL/mL of lithium bis(trifluoromethanesulfonyl)imide salt (Li-TFSI, 99.95%, Sigma Aldrich) from a stock solution of 170 mg/mL in acetonitrile. Finally, the PTAA layer was deposited using spin coating at 4000 rpm for 20 s. A low-temperature ITO layer with a thickness of 220 nm was deposited using an industrial in-line magnetron sputtering system (KENOSISTEC S.R.L., KS 400 In-Line, Milano, Italy), at a pressure of 1.1 × 10^−3^ mBar and an RF power of 90 W with inert argon gas (40 sccm). Encapsulation was achieved using a 25 mm × 25 mm, 1.1 mm-thick microscope slide coated with a UV-curable resin (3164 Three Bond, Three Bond Chemical Ltd., West Chester, PA, USA).

### Sample Characterization

XRD. The diffractograms were collected in Bragg–Brentano geometry, in the 5° < 2θ < 35° angular range (step size [°2θ] = 0.026, scan step time [s] = 555) using a Panalytical Empyrean X-ray diffractometer working with a Cu anode X-ray lamp (K-α1 [Å] = 1.54060; K-α2 [Å] = 1.54443) and a PixCel 3D detector in linear mode, setting the incident optical pathway by the fixed divergent slits (divergence slit size [°] = 0.2177). Samples were placed onto a flat sample holder for the thin films, and the generator parameters were set at 45 mA and 40 kV. In situ time-resolved measurements were performed collecting an XRD pattern each hour for 50 h during illumination with cold light.

AFM. Sample morphology was investigated by an in-house developed atomic force microscope equipped with a 30 μm × 30 μm scanner. Non-contact mode measurements were taken from several areas of the sample surface using a gold-coated ultra-sharp non-contact AFM probe (NT-MDT Spectrum Instruments, Moscow, Russia).

Photocurrent density-voltage (J–V). JV curves of the devices were collected in ambient air using a calibrated solar simulator (ABET Sun 2000, Class A, Milford, CT, USA), mimicking AM 1.5 conditions with an irradiance of 100 mW cm^−2^. Calibration was performed using a certified reference silicon cell (RERA Solutions RR-1002, Nijmegen/The Netherlands). The J–V characteristics were obtained in both forward and reverse scan directions with a scan speed of 200 mV/s and a voltage step of 20 mV, using a commercially available four-probe source-meter with multiple channels (Arkeo-Ariadne, Cicci Research srl, Grosseto, Italy).

## 3. Results and Discussion

**Figure 1:**
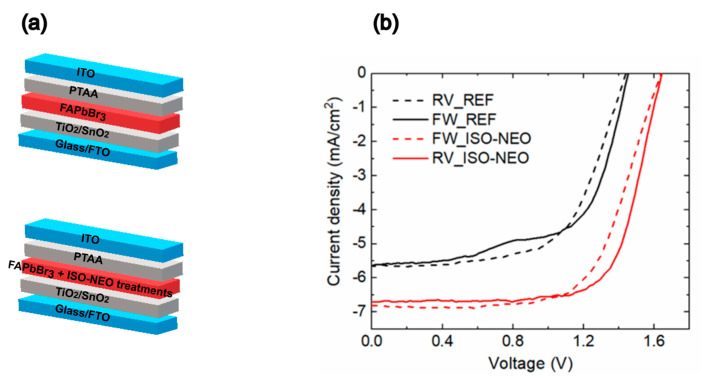
(**a**) Schematics of the solar cell stack (top: reference; bottom: passivated device) and (**b**) J–V curves of the reference and ISO-NEO devices in forward and reverse scan.

In this work, the stability of FaPbBr_3_ solar cells is investigated, focusing on the evaluation of the morpho-structural properties of the material in air under light stress [43], in correlation with the photovoltaic performances of the devices. In particular, the study is dedicated to the investigation of the role of the ISO-NEO perovskite interface passivation on the structural stability of the FAPbBr_3_ active layer and on device performance. In order to shed light on this topic, a comparative study was performed both on the reference and on ISO-NEO-passivated devices (the schematics of the solar cell stack, for both the reference and passivated device, are reported in Figure 1a). In Figure 1b and Table 1, the J–V curves and the corresponding photovoltaic (PV) parameters of the investigated reference and ISO-NEO devices are reported.

The ISO-NEO passivation strategy improved the overall PCE of the device by almost +40% (from 5.05 to 7.83). The growth of ISO-NEO on the 3D perovskite surface addresses the issue of the poor surface wettability of the PTAA solution, leading to enhancements in all PV parameters [44] (Table 1 shows the results of the PV parameters). The improvement is primarily attributed to the J_SC_ (+20%), while the V_OC_ increased by 13%, from 1.44 V to an outstanding 1.64 V, and the FF improved by 13%.

**Figure 2:**
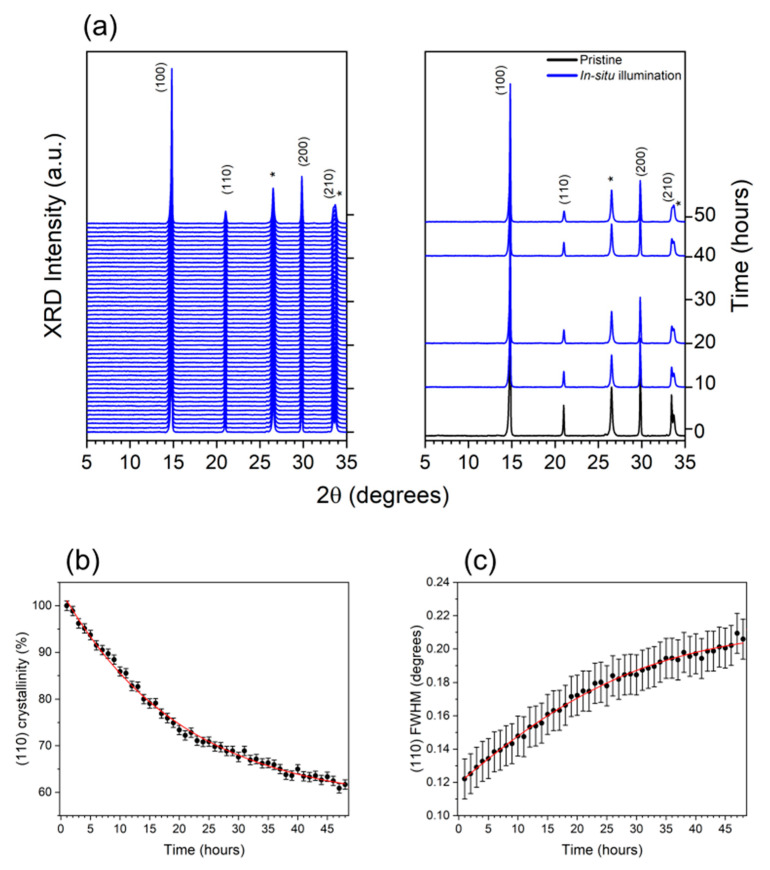
XRD analysis of the Glass/FTO/c-TiO_2_/SnO_2_/FAPbBr_3_ intermediate. (**a**) Sequence of XRD patterns collected as function of time during 48 h illumination with cold light, up to complete stability of the structural parameters (**left panel**); (**right panel**) comparison of a selection of XRD patterns collected before (black line) and during in situ illumination at different times (blue lines). FTO-characteristic reflections are labelled with an asterisk. Temporal evolution of (**b**) crystallinity and (**c**) FWHM, as evaluated by the Gaussian fitting of the (110) 3D perovskite reflection.

In situ XRD under prolonged illumination (performed for an appropriated amount of time, monitoring the structural parameter up to the conclusion of the process) and the ex situ AFM measurements were performed on the intermediate Glass/FTO/c-TiO_2_/SnO_2_/FAPbBr_3_ multi-layer and on the complete reference and ISO-NEO devices. Figure 2a summarizes the results of the in situ characterization obtained for the intermediate sample, revealing the presence of FTO-characteristic reflections (labeled with asterisks) and the perovskite 3D α-phase fingerprint (JCDD card nr. 001-0625 and [45], respectively). As is clearly visible in the right panel of Figure 2a, a crystallinity loss of the 3D α-phase perovskite can be detected during the illumination of the sample, observing the loss in intensity associated with the (110) and (210) reflections. An evaluation of the crystallinity loss of the 3D α-phase and of the grain size, obtained from the Full Width at Half Maximum (FWHM) values, was obtained by means of Gaussian fitting each perovskite reflection, as reported in Figure 2b,c. The reported results, obtained by quantitatively analyzing the (110) reflection time evolution, are representative of isotropic crystallographic behavior. Due to its orientation, the (110) reflection is representative of a direction crossing the volume of the cubic cell and the variations associated with such a plane are thus indicative of structural changes affecting the volume of the unite cell along the three lattice directions a, b and c. An overall crystallinity loss of about 40% was observed, and a characteristic time of ≈20 h was deduced from the exponential decay fitting of the trend in Figure 2b. As shown in Figure 2c, a significant increase in the (110) FWHM can also be observed during the exposure of the sample to light stress, corresponding to an overall grain size reduction from 68 ± 2 nm to 38 ± 2 nm (≈40%), as deduced by the Scherrer equations [46]. A correlation can be established between the temporal trends associated with crystallinity loss and FWHM increase, as obtained by fitting the data in Figure 2c; the latter is characterized by a time constant of ≈18 h, compatible with the characteristic time deduced for the crystallinity decay in Figure 2b. These interpretations suggest that the crystallinity loss of the 3D α-phase occurs via a grain size reduction mechanism and mainly affects larger grains (in a triangular approximation of the peak shape, its height is reduced, the base is constant and FWHM is enhanced). Since larger grains are thermodynamically more stable than the smaller ones, such experimental evidence relates the degradation mechanism to the mosaicity of the film as follows: larger crystallites indeed produce larger voids at the grain boundaries, while smaller ones allow for optimized packing. Therefore, we can conclude that the observed degradation of the FAPbBr_3_ structural properties mainly occurs at the grain boundaries, finally leading to a significant reduction in the crystalline domain size. Such a hypothesis is supported by the reported literature on FAPbI_3_, where the adsorption of water at the grain boundaries after the exposure of the material to humidity was found to be one a major source of the degradation of the perovskite α-phase [19,47]. The problem of instability at grain boundaries was found to strongly affect the performances of FAPbI_3_ solar cells and has also been pointed out for FAPbBr_3_-based devices [48]. For this reason, different synthesis processes have been proposed in order to find efficient strategies for defect passivation with the consequent beneficial effects on the device’s long-term stability and efficiency [41].

**Figure 3:**
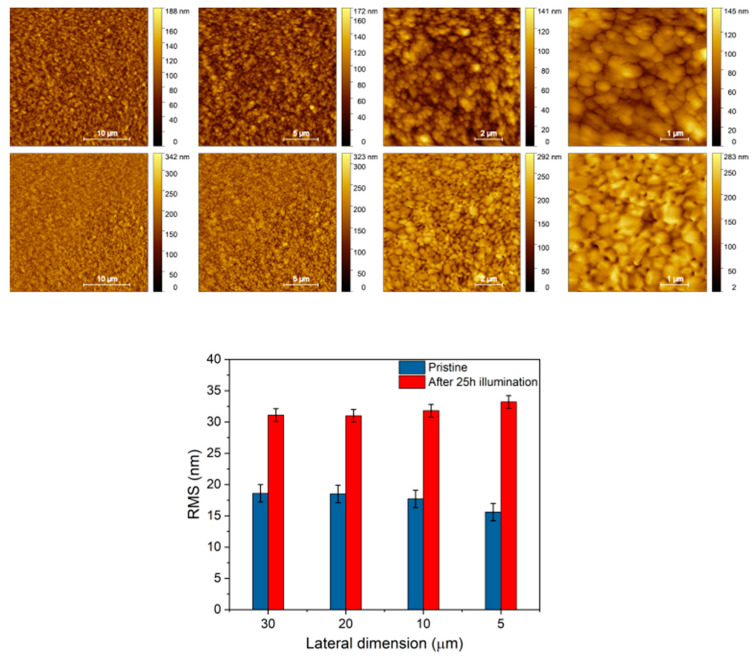
AFM analysis of Glass/FTO/c-TiO_2_/SnO_2_/FAPbBr_3_. Comparison of AFM images, ranging from the 30 μm to 5 μm lateral dimension collected before (**upper row**) and after 50 h illumination (**lower row**), and histograms showing the AFM statistical RMS analysis.

The above considerations are also corroborated by the evidence of clear morphological modifications occurring upon illumination of the intermediate device at the FAPbBr_3_ surface, as shown in the AFM images reported in Figure 3. By evaluating the surface root mean square roughness (RMS) from the collected AFM images, a significant increase in the average RMS values from 18 ± 1 nm to 32 ± 1 nm was found after light aging, as summarized in the histogram of Figure 3. The observation of an increase in the RMS after illumination supports the hypothesis that grain boundaries are primarily affected by light-induced degradation phenomena, finally leading to drastic changes in the perovskite morpho-structural properties, in agreement with the XRD results previously discussed.

**Figure 4:**
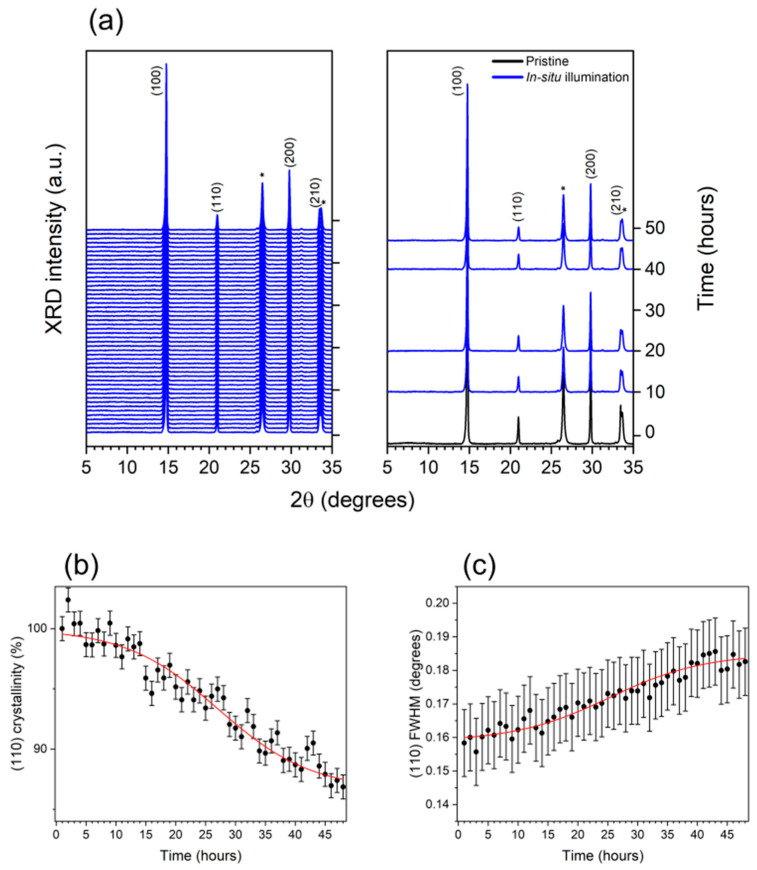
XRD analyses of Glass/FTO/c-TiO_2_/SnO_2_/FAPbBr_3_/PTAA/ITO reference device. (**a**) Temporal sequence of the XRD patterns collected under 48 h illumination with cold light, up to complete stability of the structural parameters (**right panel**); comparison of the XRD patterns of the sample before (black line) and during in situ illumination at different times (blue lines). FTO-characteristic reflections are labelled with an asterisk. Temporal evolution of (**b**) the crystallinity and (**c**) FWHM, as evaluated by the Gaussian fitting of the (110) 3D perovskite reflection.

Still, the free surface of the intermediate sample is obviously extremely exposed to ambient conditions, so conclusions regarding the structural degradation affecting the FAPbBr_3_ active layer, incorporated in a closed device, cannot be straightforward. Therefore, in order to gain direct evidence of the role of grain size on the structural stability of FAPbBr_3_, a complete reference device was investigated under the same experimental conditions as above. The results of the in situ XRD measurements, carried out on the Glass/FTO/c-TiO_2_/SnO_2_/FAPbBr_3_/PTAA/ITO multi-layer reported in Figure 4a, reveal a distinct kinetic trend compared to the intermediate sample presented before. As shown in Figure 4b, the perovskite crystallinity loss in the latter case is limited to about 15%, with a characteristic time of ≈24 h, as estimated by the sigmoidal fitting of the trend in Figure 4b. A small grain size reduction from 53 ± 2 nm to 45 ± 2 nm (about 15%) is observed from the temporal evolution of the (110) FWHM in Figure 4c, showing a characteristic time of ≈22 h, as deduced by the sigmoidal fitting of the data. Furthermore, the saturation time estimated from the trends in Figure 4b,c was found to be around 34 h for both processes, thus suggesting that the decrease in the (110) crystallinity can be directly correlated to a grain size reduction, as already observed for the Glass/FTO/c-TiO_2_/SnO_2_/FAPbBr_3_ sample. Nevertheless, compared to the intermediate device, a slower kinetic of degradation, characterized by an induction time, was found in this case. Such observations suggest that the PTAA hole transporting layer (HTL)and the ITO electrode can act as barrier layers limiting and delaying the FAPbBr_3_ degradation process, therefore suggesting that exposure to ambient conditions (in particular, relative humidity) strongly contributed to immediately triggering crystallinity loss, when the intermediate sample was studied. However, despite the protective role of the PTAA and ITO layers, such phenomenon is still observed in the full device stack, triggered at the grain boundaries, although much less effective and delayed by an induction time. This is a hint that degradation factors arising from external ambient conditions have been significantly limited, closing the device, but the perovskite film is still not completely stable.

**Figure 5:**
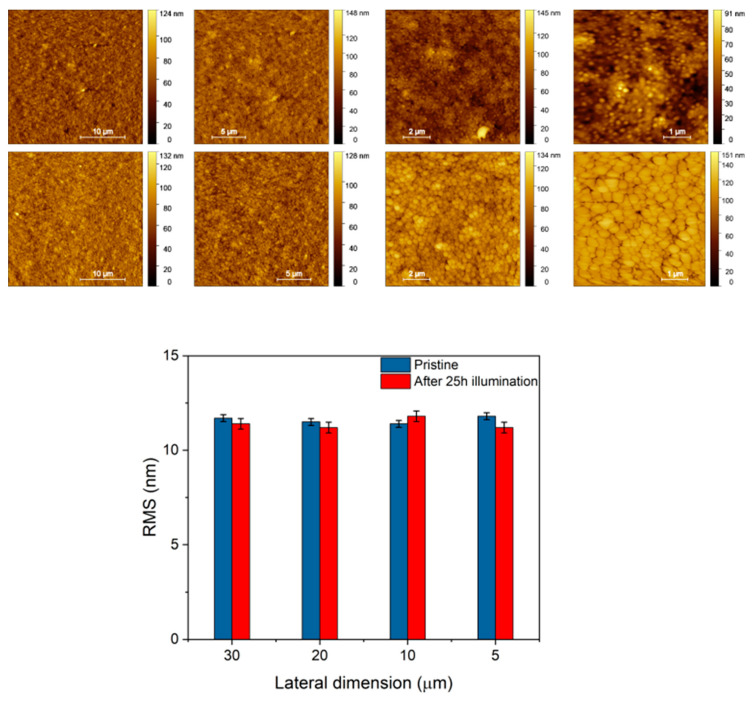
AFM analysis of reference Glass/FTO/c-TiO_2_/SnO_2_/FAPbBr_3_/PTAA/ITO device. Comparison of AFM images of the ITO surface, collected before (**upper row**) and after (**lower row**) 48 h illumination with cold light, and histograms showing the AFM statistical analysis.

The morphological characterization of the Glass/FTO/c-TiO_2_/SnO_2_/FAPbBr_3_/PTAA/ITO, summarized in Figure 5, reveals the presence of globular structures, typical of the ITO. As estimated from the AFM analysis reported in the histogram in Figure 5, surface homogeneity is generally very high and essentially unaffected by the aging process, validating the barrier role of the ITO electrode. The RMSs evaluated before and after illumination are comparable, with average values of 11.6 ± 0.3 nm and 11.4 ± 0.3 nm, respectively.

**Figure 6:**
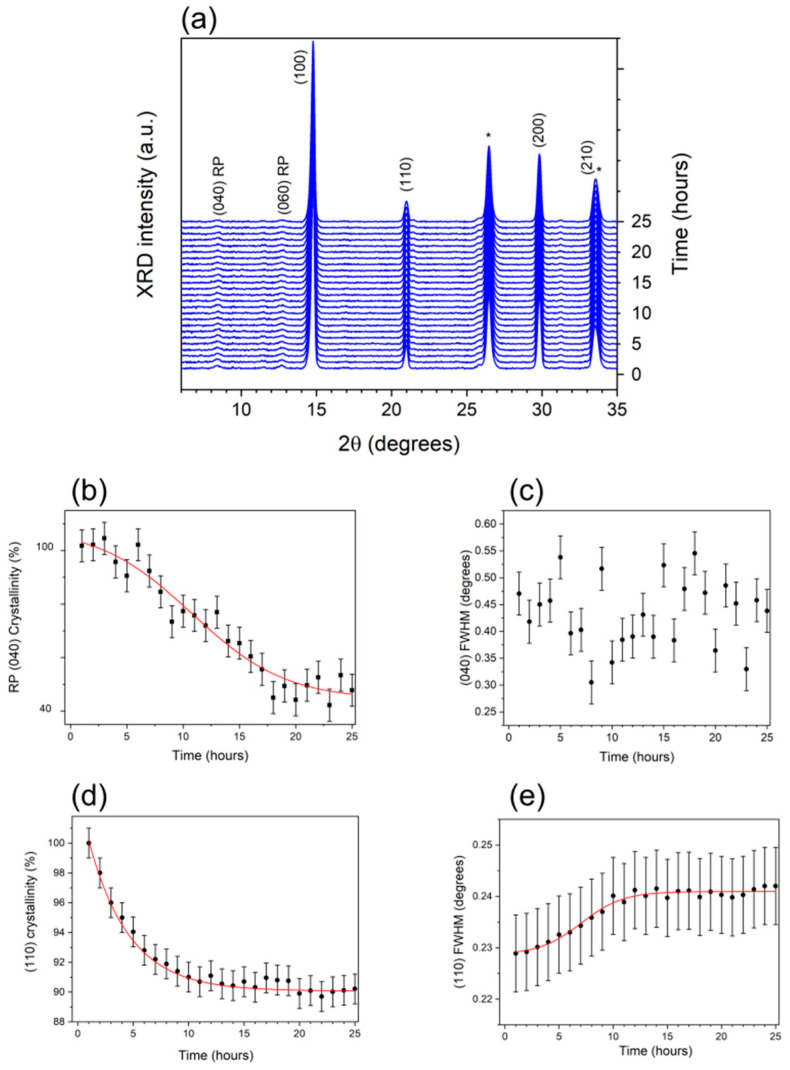
XRD analysis of Glass/FTO/c-TiO_2_/SnO_2_/FAPbBr_3_/ISO-NEO/PTAA/ITO device. (**a**) Temporal sequence of the XRD patterns collected upon illumination with cold light, conducted to complete the stability of the structural parameters (25 h). FTO-characteristic reflections are labelled with an asterisk. Temporal evolution of (**b**) crystallinity, (**c**) FWHM of the 2D phase and (**d**) crystallinity. (**e**) FWHM, as evaluated by the Gaussian fitting of the (110) 3D perovskite reflection (from the patterns in (**a**)).

Since the complete reference device still showed small but non-negligible perovskite crystallinity loss, in situ time-resolved XRD measurements were finally performed on the Glass/FTO/c-TiO_2_/SnO_2_/FAPbBr_3_/ISO-NEO/PTAA/ITO to evaluate the role of ISO-NEO on the morpho-structural stability of FAPbBr_3_-based solar cells. The results obtained are shown in Figure 6a, where the sequence of XRD patterns, collected as a function of time and 2θ, are presented. In addition to the 3D perovskite and FTO reflections, other features are also observed at 2θ = 4.23° (and multiples). We can ascribe these features to the (0k0) lattice planes of 2D RP perovskite structure [49,50]. This growth is strongly favored when the number of layers is more than 1, and we can assume by the peak position that the number of layers is approximatively between 2 and 4. This is an indication that the ISO-NEO layer on top of the perovskite film intercalates, inducing the formation of the 2D phase, which is known to act as a protective/stabilizing phase in perovskite-based devices [41,51]. The Gaussian fitting procedure of the RP reflections was adopted, and the results in Figure 6b, relative to the crystallinity loss of the (040) RP reflection, are representative of the whole behavior of the 2D phase. A visible overall 70% crystallinity loss of the 2D phase is observed, with a characteristic time of 11 h, as deduced by the sigmoidal fit (red line) of the trend. On the contrary, the evaluation of the 3D α-phase crystallinity revealed an almost negligible (less than 10%) crystallinity loss, with characteristic time of ≈4 h, as estimated by the exponential decay fitting of the trend in Figure 6d. It is worth noting that such an evaluation could be performed only due to the high precision of the in situ measurements, detecting any minimal crystallinity loss. A very limited 3D α-phase grain size reduction was also observed. It is important to stress how, despite the overall reduction in the grain size in the ISO-NEO sample after 48 h illumination being quantitatively negligible, the accuracy of the in situ approach still allows for identifying a growing trend in the temporal evolution of the FWHM, with characteristic time of ≈7 h and saturation time of about 11 h, as deduced by the sigmoidal fitting of the data in Figure 6e.

By comparing the plots in Figure 6b,d different kinetics can be identified for the two processes as follows: while the (110) crystallinity is immediately affected by the light stress, showing a decrease within the first 4 h of illumination, the RP (040) crystallinity loss begins after an initial latency of approximatively 4 h, reaching its maximum speed after about 11 h. Interestingly, the time corresponding to the maximum variation rate of the 3D phase crystallinity is compatible with the induction time required to trigger the crystallinity loss in the 2D phase. Furthermore, the saturation time estimated from the kinetic of the 3D phase crystallinity (about 10 h) compares well with the time at which the maximum variation rate of the 2D phase crystallinity is observed. By comparing the characteristic times of the two processes, we can conclude that the loss in crystallinity of the 2D phase occurs consequently to that of the 3D phase. It is thus reasonable to suppose that the decrease in crystallinity of the 3D phase, which is much limited in this case compared to the untreated reference device (see Figure 4b), can trigger the structural changes observed in the 2D phase. Such 3D degradation affects the 2D layer locally, inducing its crystalline to amorphous conversion but, importantly, it is able to limit the propagating aging effect within the perovskite bulk.

Despite the loss in crystallinity associated with the (040) RP reflection being quite significant, no evidence of variation in coherence length along the z-axes of the 2D phase is found, as deduced from the (040) FWHM temporal evolution in Figure 6c. It is thus likely that the changes affecting the crystallinity of the 2D phase are very spatially localized, presumably at the interface with the 3D phase, where a higher density of defects is expected to occur [52]. Due to their high affinity for oxygen and water molecules, vacancies and point defects are known to be among the major sources of instability, accelerating the perovskite degradation process via the vacancy-assisted decomposition mechanism [53]. At this stage, the question is how the enhanced structural stability of the ISO-NEO device is reflected on the aging PV device.

**Figure 7:**
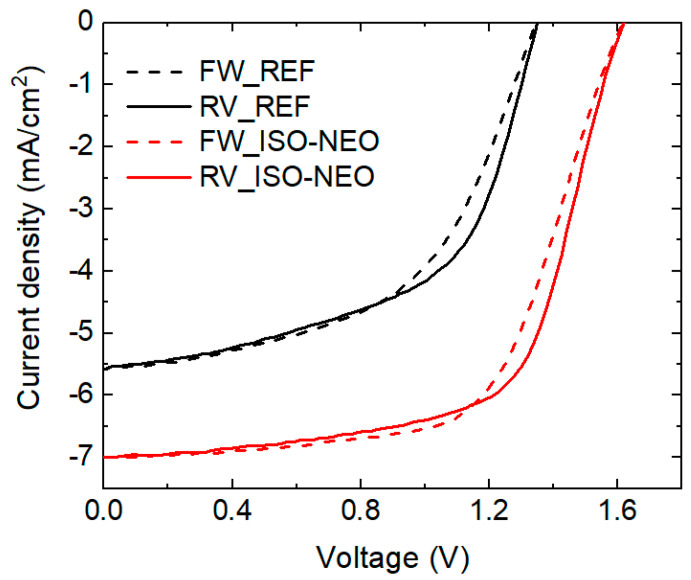
J–V curves of the reference and ISO-NEO device in the forward and reverse scan after 24 h of light soaking aging.

In Figure 7 and Table 2, the J–V curves and the corresponding PV parameters of the investigated reference and ISO-NEO devices after 24 h of light soaking are reported. Following light soaking stress, the degradation rate for the reference device is 18%, while for the ISO-NEO device it is 7%. The REF device indeed exhibited a clear burn-in degradation, a common behavior in perovskite and organic solar cells, for which several mechanisms have been proposed [54,55].

In our case, the origin of the burn-in is likely located at the interface between PTAA and the perovskite [44,54]; therefore, the introduction of the ISO passivator represents a winning strategy for improving PV stability as well as the morpho-structural stability.

The above observations validate the results discussed above, highlighting the importance of the ISO-NEO passivating layer in order to preserve cell stability towards the extrinsic ambient factors affecting the active perovskite material.

**Figure 8:**
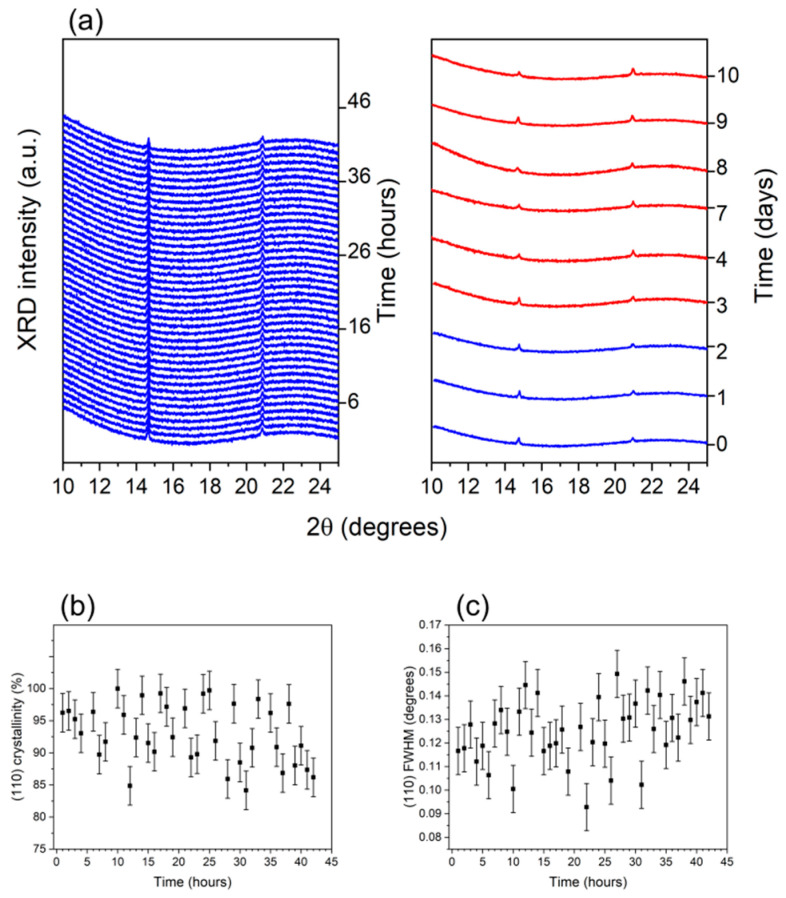
XRD analysis of the Glass/FTO/c-TiO_2_/SnO_2_/FAPbBr_3_/ISO-NEO/PTAA/ITO encapsulated cell. (**a**) Sequence of the in situ XRD patterns collected with the device as a function of time during illumination with cold light for 42 h, up to complete stability of the structural parameters (**left panel**). Sequence XRD patterns collected on the device submitted to continuous illumination for an overall illumination time of 10 days (**right panel**); ex situ XRD patterns (red lines) are compared to the in situ spectra of device in the pristine state, after 24 h and 42 h (blue lines); Temporal evolution of (**b**) crystallinity and (**c**) FWHM as evaluated by the Gaussian fitting of the (110) 3D perovskite reflection during in situ 42 h illumination with cold light of the encapsulated device.

As shown, the most important factor triggering the degradation process appears to be an extrinsic factor arising from the adsorption of water occurring when the cell is exposed to environmental conditions (due to the partial covering of the cell top surface by the electrode or the fact that it is laterally exposed to air) trapped at the grain boundaries. Still, in order to definitively exclude the presence of additional concomitant intrinsic factors (such as water inopportunely residual from the deposition process) that may also contribute to the observed instabilities, in situ XRD measurements were performed on a Glass/FTO/c-TiO_2_/SnO_2_/FAPbBr_3_/ISO-NEO/PTAA/ITO-encapsulated device. The obtained results are summarized in the left panel of Figure 8a, where the XRD sequence, collected as a function of time and 2θ, is reported. As visible, despite the presence of the encapsulating agent, a reduction in the intensity associated with the perovskite reflections is observed, and the (100) and (110) features of the 3D phase were still clearly detectable. As shown in Figure 8b,c, the quantitative analysis performed by the Gaussian fitting of the (110) signal revealed that the encapsulated sample is essentially stable, the dispersion of the crystallinity and FWHM experimental data being symmetric and not showing any trend (different from what was observed for the previous samples). Indeed, both the (110) crystallinity and FWHM are not affected by prolonged illumination (42 h), thus confirming that the 3D perovskite phase is well preserved when encapsulation is carried out. To further obtain insights into long-term stability, after the in situ characterization, the encapsulated device was continuously kept under illumination and an ex situ XRD pattern was collected to follow the evolution of the (100) and (110) reflections. As shown in the right panel of Figure 8a, where the results of this ex situ XRD characterization are summarized, the encapsulated device is structurally stable during continuous illumination.

It is thus reasonable to conclude that extrinsic factors are the main source of degradation limiting the stability of the ISO-NEO device.

## 4. Conclusions

In this work, the structural and morphological properties and stability of PSCs based on FAPbBr_3_ solar cells under working conditions are investigated. Insights are gained into the influence of the interface passivation of the active perovskite material, allowing us to enhance PV parameters, via a comparative study on both a reference and ISO-NEO-passivated complete devices, as well as on the intermediate multi-layer Glass/FTO/c-TiO_2_/SnO_2_/FAPbBr_3_. The growth of ISO-NEO on the 3D perovskite leads to enhancements in all PV parameters. Due to the combined use of in situ XRD and ex situ AFM, the structural and morphological modifications occurring under exposure to light stress were monitored and correlated with the photovoltaic performances of the devices. It is evidenced that ISO-NEO contributes to the improved operational stability of PSCs in comparison to the reference device, by passivating defects at the grain boundaries and interfaces within the perovskite layer. From the morpho-structural characterization of the samples, the degradation processes occurring at the grain boundaries were deduced, with the passivated ISO-NEO device showing an increased stability compared to the reference sample. Our experimental approach allowed us to identify a very limited degradation for the ISO-NEO sample (characterized by a slower kinetics), revealing the role of 2D passivation on the stability of FAPbBr_3_. A correlation was established between the crystallinity loss of the 3D and 2D phases as follows: our results point out that the crystallinity loss in the 2D phase occurs consequentially to the crystallinity loss of the 3D phase. The structural modifications associated with the 2D phase were spatially localized, evidencing that the observed degradation processes are triggered at the 2D–3D interface, where a higher concentration of defects is expected. Such defects can act as a source of instability, being the preferred site for water uptake in ambient conditions observed in the reference device (and much limited, though not completely inhibited, in the ISO-NEO device). Finally, the study performed on an encapsulated cell allowed us to exclude the presence of the additional concomitant intrinsic aging factor, as follows: when the device is encapsulated, the perovskite layer is fully preserved and the a-phase does not showing any structural modification throughout the investigated period (overall illumination in air up to 10 days).

## Figures and Tables

**Table 1:** PV parameters from the J–V curves of the reference and ISO-NEO devices shown in Figure 1.

	Scan	V_OC_ (V)	J_SC_ (mA/cm^2^)	FF (%)	PCE (%)
Reference	FW	1.44	5.61	61.33	4.95
	RV	1.45	5.63	61.98	5.05
ISO-NEO	FW	1.63	6.81	64.94	7.25
	RV	1.64	6.69	71.03	7.83

**Table 2:** PV parameters from the J–V curves of the reference and ISO-NEO devices shown in Figure 7.

	Scan	V_OC_ (V)	J_SC_ (mA/cm^2^)	FF (%)	PCE (%)
Reference	FW	1.34	−5.58	52.86	3.97
	RV	1.35	−5.56	55.55	4.17
ISO-NEO	FW	1.61	−7.00	62.59	7.08
	RV	1.62	−7.00	64.40	7.31

## Data Availability

The data supporting the findings of this study are available from the corresponding author upon reasonable request.

## Abbreviations

The following abbreviations are used in this manuscript:

PSCs	Perovskite solar cells
FAPbBr_3_	Formamidinium lead bromide
PTAA	Poly[bis(4-phenyl)(2,4,6-trimethylphenyl)amine]
Glass/FTO	Fluorine-doped tin oxide glass
ITO	Indium tin oxide
ISO	iso-Pentylammonium chloride
NEO	neo-Pentylammonium chloride
XRD	X-ray diffraction
AM	Atomic force microscopy
BIPV	Building integrated photovoltaics
MAPbI_3_	Methylammonium lead iodide
FAPbI_3_	formamidinium lead iodide
PCE	Power conversion effficiency
PEABr	Phenetylammonium bromide
BMIMBF4	1-Butyl-3-methylimidazolium tetrafluoroborate
IL	Ionic liquid
c-TiO_2_	TiO_2_ compact layer
TBP	4-tert-butyl pyridine
Li-TFSI	Lithium bis(trifluoromethanesulfonyl)imide salt
PV	Photovoltaic
